# Assessing Instructional Cognitive Load in the Context of Students' Psychological Challenge and Threat Orientations: A Multi-Level Latent Profile Analysis of Students and Classrooms

**DOI:** 10.3389/fpsyg.2021.656994

**Published:** 2021-07-01

**Authors:** Andrew J. Martin, Paul Ginns, Emma C. Burns, Roger Kennett, Vera Munro-Smith, Rebecca J. Collie, Joel Pearson

**Affiliations:** ^1^University of New South Wales, Kensington, NSW, Australia; ^2^The University of Sydney, Sydney, NSW, Australia; ^3^Macquarie University, Sydney, NSW, Australia; ^4^The King's School, North Parramatta, NSW, Australia

**Keywords:** cognitive load, load reduction instruction, cognitive appraisal, engagement, achievement, latent profile analysis, multilevel, construct validity

## Abstract

To better understand instructional cognitive load, it is important to operationalize and assess it in novel ways that can reveal how different students perceive and experience this load as either challenging or threatening. The present study administered a recently developed instruction assessment tool—the Load Reduction Instruction Scale-Short (LRIS-S)—to *N* = 2,071 students in 188 high school science classrooms. Multilevel latent profile analysis (LPA) was used to identify student and classroom profiles based on students' reports of instructional cognitive load (load reduction instruction, LRI; using the LRIS-S) and their accompanying psychological challenge orientations (self-efficacy and growth goals), and psychological threat orientations (anxiety and failure avoidance goals). In phase 1 of analyses (investigating students; Level 1), we identified 5 instructional-psychological student profiles that represented different presentations of instructional load, challenge orientation, and threat orientation, ranging from the most maladaptive profile (the Instructionally-Overburdened & Psychologically-Resigned profile) to the most adaptive profile (Instructionally-Optimized & Psychologically-Self-Assured profile). The derived profiles revealed that similar levels of perceived instructional load can be accompanied by different levels of perceived challenge and threat. For example, we identified two profiles that were both instructionally-supported but who varied in their accompanying psychological orientations. Findings also identified profiles where students were dually motivated by both challenge and threat. In turn, these profiles (and their component scores) were validated through their significant associations with persistence, disengagement, and achievement. In phase 2 of analyses (investigating students and classrooms; Levels 1 and 2), we identified 3 instructional-psychological classroom profiles that varied in instructional cognitive load, challenge orientations, and threat orientations: Striving classrooms, Thriving classrooms, and Struggling classrooms. These three classroom profiles (and their component scores) were also validated through their significant associations with classroom-average persistence, disengagement, and achievement—with Struggling classrooms reflecting the most maladaptive outcomes and Thriving classrooms reflecting the most adaptive outcomes. Taken together, findings show that considering instructional cognitive load (and new approaches to empirically assessing it) in the context of students' accompanying psychological orientations can reveal unique insights about students' learning experiences and about important differences between classrooms in terms of the instructional load that is present.

## Introduction

In this study, we propose that instructional cognitive load is likely to be perceived and experienced in different ways by different students. Following cognitive appraisal theory (e.g., Lazarus and Folkman, 1984; for reviews, see Roseman and Smith, 2001; Moors et al., 2013), we suggest that some students will perceive cognitive load in an approach- and challenge-oriented way, while other students will perceive cognitive load in an avoidant- and threat-oriented way. This being the case, we suggest that we can better understand instructional cognitive load when we consider it in the context of students' accompanying psychological challenge and threat orientations. The present study does so from a person-centered perspective (using latent profile analysis; LPA) based on students' reports of instructional load (load reduction instruction, LRI; using the Load Reduction Instruction Scale-Short, LRIS-S) and their accompanying psychological challenge orientations (self-efficacy and growth goals) and psychological threat orientations (anxiety and failure avoidance goals).

In addressing these issues, we adopt a *construct validation* approach. Researchers in educational psychology have long emphasized the importance of evaluating instruments within a construct validation framework (e.g., Marsh, 2002; Martin, 2007, 2009). Construct validation can be classified in terms of *within-network* and *between-network* approaches. Within-network approaches explore the internal structure of a construct or network and between-network approaches typically seek to establish a logical, theoretically meaningful pattern of relations between constructs and networks. Both approaches tend to employ variable-centered analyses such as reliability and factor analysis (for within-network validity) and correlation and regression (for between-network validity). Indeed, this study's central measurement tool (the Load Reduction Instruction Scale; LRIS) has been validated using these variable-centered within- and between-network approaches (Martin and Evans, 2018).

**Figure 1:**
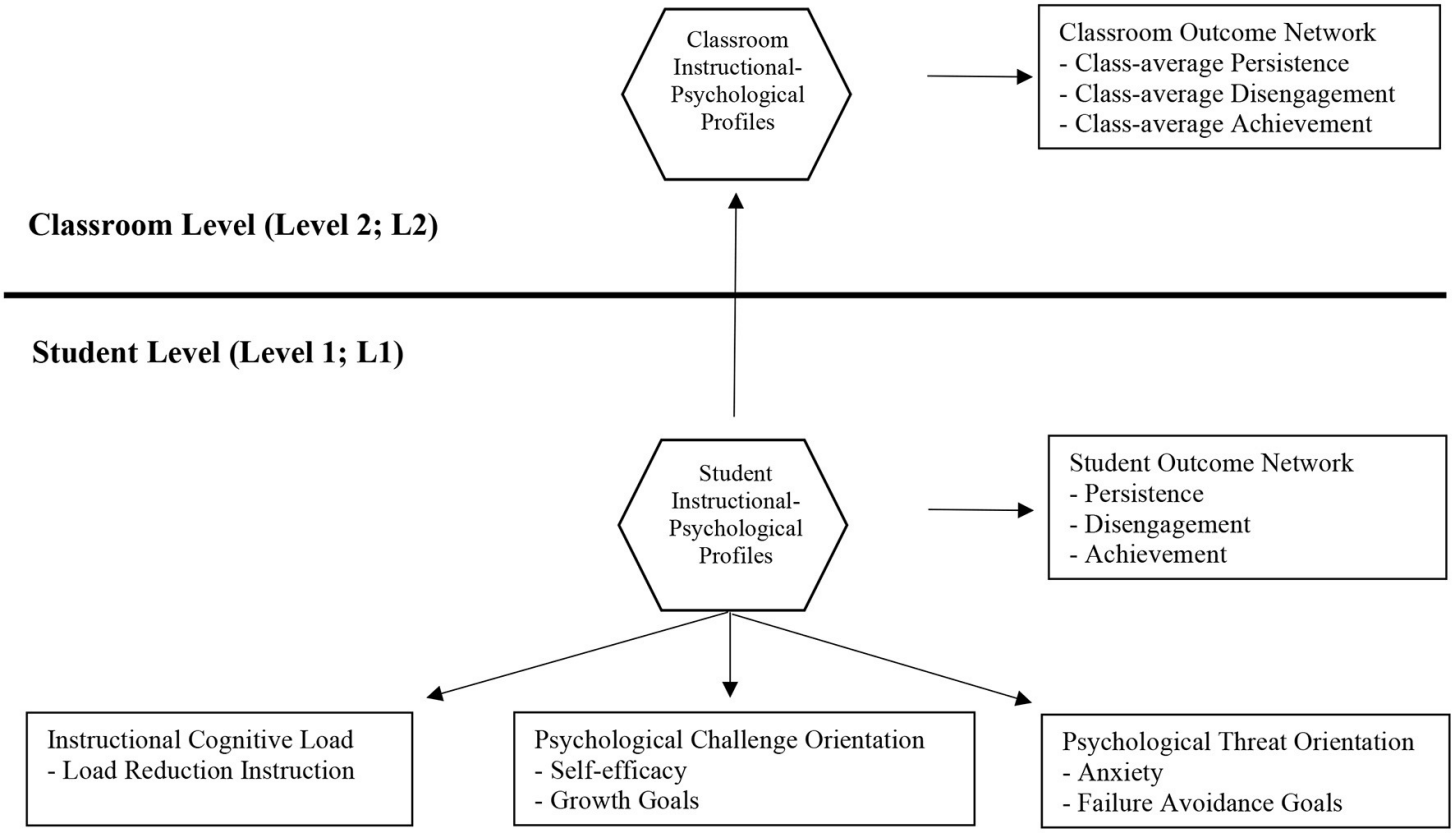
Hypothesized models tested in the study at the student- and classroom-level.

However, as discussed below, variable-centered approaches to construct validation can mask important phenomena among subpopulations of the wider population. Person-centered approaches, on the other hand, are well-placed for validation at a more granular subpopulation level. Therefore, the present investigation applies a person-centered approach to the assessment of within- and between-network validity. Using multilevel LPA and integrating and synthesizing cognitive load theory (Sweller, 2012) with cognitive appraisal theory (Lazarus and Folkman, 1984), we test within-network validity by identifying a network of theoretically plausible student and classroom profiles that are based on student reports of instructional load (via the LRIS) and students' accompanying psychological challenge and threat orientations. We then test between-network validity by exploring the links between the network of student and classroom profiles and a theoretically plausible network of outcome variables (persistence, disengagement, achievement). In essence, then, the present study contributes a novel multilevel construct validity approach to person-centered analysis in a bid to understand the nomological network of instructional cognitive load. Figure 1 shows the hypothesized model.

This approach to construct validity draws on guidelines advanced by methodologists in the measurement space—very much inspired by the seminal work of Campbell and Fiske (1959). Psychological research (including educational psychology research) typically involves hypothetical constructs that are unobservable, conceptual, or theoretical abstractions (Marsh et al., 2006). Constructs are often inferred indirectly via observable indicators. A vital question, then, is how well these indicators represent the hypothetical construct, including the extent to which the construct is: well-represented by derived scores, is well-defined, and is related to variables to which it should be theoretically connected (Marsh, 2002; Marsh et al., 2006). In light of these critical questions, it is recommended that construct validity research comprises multiple perspectives based on multiple methods. According to Marsh et al. (2006), this involves the use of “multiple indicators of each construct, multiple constructs and tests of their a priori relations, multiple outcome measures, multiple independent/manipulated variables, multiple methodological approaches … the multiple perspectives provide a foundation for evaluating construct validity based on appropriate patterns of convergence and divergence and for refining measurement instruments, hypotheses, theory, and research agendas” (p. 442). Accordingly, the present construct validation study includes latent factors (comprised of multiple indicators), multiple factors and tests of their relations, numerous outcome measures, and integration of two analytical methods by way of LPA and multi-level modeling. In these ways, we claim to provide a unique perspective on multilevel construct validity.

## Cognitive Load Theory

Cognitive load theory has identified principles of instruction that are aimed at easing the cognitive burden on students as they learn (Sweller et al., 2011; Sweller, 2012). According to cognitive load theory, there are two kinds of cognitive load that can be imposed during instruction and that impede learning: intrinsic and extraneous load (Sweller et al., 2011). Intrinsic cognitive load is a function of the inherent complexity of instructional activity and material, given the learner's prior knowledge. Teachers can manage intrinsic cognitive load by presenting instructional material that is appropriate to the level of knowledge of students (Sweller et al., 2011). Extraneous cognitive load emanates from the way that instructional material is structured and presented (Sweller et al., 2011). Teachers can manage this latter form of load by presenting instructional material sequentially, clearly, and explicitly to students; in doing so, students are guided through learning in a structured and linear fashion, leading to low extraneous load. However, extraneous load is high when instructional material is presented such that students need to figure out the informational structure, have to decide between a range of potential solutions, and/or apply information about which they have low prior knowledge (Sweller et al., 2011). Extraneous cognitive load is identified as an unnecessary burden on students as it does not contribute to learning (Sweller et al., 2011).

To date, the bulk of research into cognitive load theory has been experimental, with cognitive load induced through presentation and manipulation of instructional/learning material to elicit conditions of low or high cognitive load (see Sweller, 2012 for a review). There is significant research assessing cognitive load through self-reports of cognitive burden (e.g., Leppink et al., 2013; Krell, 2017), and other approaches such as through electrodermal activity, neurological activity, eye tracking, blood flow, physical pressure exerted on a computer mouse, etc. (e.g., Paas et al., 1994; Ikehara and Crosby, 2005; Wang et al., 2014; Howard et al., 2015; Ghaderyan et al., 2018). This research has assessed the presence of cognitive load, as well as the instructional techniques aimed at managing or reducing cognitive load. Much of this research has taken place under experimental conditions. This experimental work has significant internal validity and has been critical to rigorously measuring cognitive load and identifying some major effects that are now well-established in the cognitive load tradition (e.g., expertise reversal effect, modality effect, split attention effect, etc.). Alongside this experimental work it is also important to extend assessment to attend to other matters of validity—such as conducting classroom-based assessment research that has the potential to inform the ecological validity of cognitive load assessment. As one significant step in this direction, recent work has articulated a multi-factor instructional framework—load reduction instruction (LRI)—aimed at (a) reducing instructional cognitive load on learners, (b) developing a multi-factor survey instrument to assess this instructional framework, and (c) administering and assessing this instrument in the classroom context (Martin, 2016; Martin and Evans, 2018, 2019; Martin et al., 2020a).

## Load Reduction Instruction (LRI) and Its Measurement

Harnessing key cognitive load theory principles, Martin (2016; see also Martin and Evans, 2018, 2019) proposed LRI as an instructional means to manage the cognitive burden students can experience as they learn. According to cognitive load theory, there is a need for instruction that accommodates the reality of the limits of working memory and helps students transfer knowledge between working and long-term memory (Paas et al., 2003; Sweller, 2004). A key means by which this can occur is by developing students' fluency and automaticity in skill and knowledge. This frees up working memory resources, reduces cognitive burden, and better enables students to transfer novel information into long-term memory (Rosenshine, 2009). Based on the Martin (2016) LRI framework, fluency and automaticity are developed through its first four principles: (principle #1) reducing the difficulty of instruction in the initial stages of learning, as appropriate to the learner's level of prior knowledge (see also Pollock et al., 2002; Mayer and Moreno, 2010); (principle #2) providing appropriate support and scaffolding to learn the relevant skill and knowledge (see also Renkl and Atkinson, 2010; Renkl, 2014); (principle #3) allowing sufficient opportunity for practice (see also Purdie and Ellis, 2005; Rosenshine, 2009; Nandagopal and Ericsson, 2012); and (principle #4) providing appropriate feedback-feedforward (combination of corrective information and specific improvement-oriented guidance) as needed (see also Shute, 2008; Hattie, 2009; Mayer and Moreno, 2010). Through these four principles, students develop fluency and automaticity and are then well-positioned to apply their skill and knowledge more independently—including through novel tasks, higher order reasoning and thinking, problem solving, and guided discovery (Martin, 2016; Martin and Evans, 2019)—which is important to guard against potential expertise reversal effects (Kalyuga et al., 2012; Chen et al., 2017). This represents principle #5: guided independent learning.

Having articulated the five key principles of LRI (Martin, 2016), Martin and Evans (2018) developed a novel tool, the Load Reduction Instruction Scale (LRIS), to administer to students to report on the instructional practices of their teacher. The LRIS comprises five factors to assess the five LRI principles (difficulty reduction, support and scaffolding, practice, feedback-feedforward, guided independence). Each factor is composed of five items (yielding a 25-item instrument). In the first empirical study of the LRIS, students in 40 high school mathematics classrooms completed the instrument (Martin and Evans, 2018). Findings revealed the scores of each factor to be reliable, the factor structure to be sound, and significant bivariate correlations with intrinsic and extraneous cognitive load in predicted directions. In a follow-up investigation that linked the Martin and Evans (2018) data with a previous survey, results showed that LRI was associated with gains in mathematics motivation, engagement, and achievement (Evans and Martin, Submitted). In another study, Martin et al. (2020a) introduced and explored a brief form of the LRIS (the LRIS-Short; LRIS-S) that was designed to capture a unidimensional latent factor in keeping with the higher order LRIS factor in the Martin and Evans (2018) research. Martin et al. (2020a) used this LRIS-S in a multilevel study of more than 180 science classrooms, finding that the link between LRI in science and science achievement (at student- and classroom-levels) was mediated by class participation, future aspirations, and enjoyment in science. However, all these studies (including their construct validity aspects) have been variable-centered. As is now discussed, to even better understand the nomological network of LRI, a construct validity approach from a multilevel person-centered perspective has much to offer.

## Person-Centered and Multilevel Assessment

### Person-Centered Assessment

As noted above, the bulk of research assessing cognitive load in students' academic lives (including LRI research) has been variable-centered. Variable-centered approaches aim to assess relations between factors across individuals (Collie et al., 2020). This has been important for conducting classic construct validity work with cognitive load factors and for identifying what cognitive load factors predict or are predicted by other factors (e.g., Leppink et al., 2013; Klepsch and Seufert, 2020). However, variable-centered approaches can mask important phenomena among subpopulations of the wider population. In contrast, person-centered research utilizes the factors in a study to identify distinct subpopulations (or profiles) of individuals (Bauer and Curran, 2004; Collie et al., 2015; Morin et al., 2017). For example, different subpopulations of students may reflect different patterns in how LRI manifests and relates to other educational factors in their classroom and academic experience. Thus, LRI may not function in a similar way for all students. To the extent this is the case, it is important to assess LRI to capture distinct subpopulations of students, if such subpopulations exist. This will generate practical yields in identifying specific student profiles that teachers can attend to in their pedagogy.

Person-centered analyses (in this investigation, latent profile analysis—LPA) are ideal for addressing these issues. Specifically, by revealing the way LRI co-occurs with other factors among subpopulations of students, person-centered assessment helps identify the different types of students that reside within the classroom and the distinct ways LRI manifests in these students' academic lives. As we describe below, we seek to further assess the construct validity of LRI from a person-centered perspective by including psychological challenge and threat orientation measures alongside LRI measures to better understand the nomological network of instructional-psychological profiles. LPA enables us to see whether there might be subpopulations of students with similar LRI levels, but who differ on psychological factors (and vice versa). As we describe below, it is theoretically plausible that students who have different psychological challenge and threat orientations will experience instructional load in different ways and our person-centered construct validity approach seeks to confirm this. The LPA represents the within-network validity aspect of our study by way of its assessment of a target network of instructional-psychological profiles.

### Multilevel Assessment

It is also the case that the bulk of cognitive load research is almost exclusively conducted at the student-level, without appropriate regard for the classrooms to which the students belong (however, LRI research is an exception—described below). There is now broad recognition of the importance of analyzing hierarchical data in appropriate ways (Marsh et al., 2012), especially when the variables of interest include references to class-wide phenomena, such as instruction (as LRI does). In single-level research designs there are statistical biases (e.g., within-group dependencies; confounding of variables within and between groups) and multilevel analyses seek to resolve biases like these (see Raudenbush and Bryk, 2002; Goldstein, 2003; Marsh et al., 2009). Multilevel analysts have identified the reciprocity of group and individual dynamics: the group can affect the individuals and individuals can affect the group (Raudenbush and Bryk, 2002; Goldstein, 2003; Marsh et al., 2009). To date, most LRI research has recognized this and conducted multilevel analyses at student- and classroom-levels—indeed, demonstrating the validity of LRI at student- and classroom-levels (Martin and Evans, 2018, Evans and Martin, Submitted; Martin et al., 2020a). But, as noted, these studies have involved variable-centered analyses, which may mask differences between subpopulations of students and classrooms. The present study extends this research by conducting multilevel LPA that not only identifies student instructional-psychological profiles, but also classroom instructional-psychological profiles. Given LRI is about classroom instruction, its construct validity must be reflected in theoretically logical classroom profiles.

## LRI and Accompanying Psychological Orientations

As described earlier, it is plausible that cognitive load will be perceived and experienced by students in different ways. Theories of cognitive appraisal (e.g., Lazarus and Folkman, 1984; for reviews, see Roseman and Smith, 2001; Moors et al., 2013) suggest that when presented with a task, an individual subjectively appraises its demands and their capacity to meet the demands. The individual perceives challenge when they believe they can meet the demands; the individual perceives threat when they believe they cannot meet the demands (see also Putwain and Symes, 2014, 2016, Uphill et al., 2019). Thus, when cognitive load is unacceptably high there may be a greater likelihood that anxiety and fear are present, and when cognitive load is effectively managed, more positive appraisals occur. At the same time, it is also the case that different students can appraise the same stimuli in different ways. For example, following cognitive appraisal theory (Lazarus and Folkman, 1984), some students will perceive cognitive load in an approach- and challenge-oriented way, while other students will perceive cognitive load in an avoidant- and threat-oriented way. Indeed, recent reviews of challenge and threat suggest that there may even be the dual presence of both challenge and threat (Uphill et al., 2019; see also Rogat and Linnenbrink-Garcia, 2019 for dual goals under approach-avoidance goal frameworks)—for example, in the event of cognitive load there may be students who perceive it as a challenge and opportunity for learning, but who also fear failing and see it as a potential threat. This idea had been previously raised by Covington (2000) and Martin and Marsh (2003) in the form of “overstrivers” who are students investing effort (in a challenge-like way), but driven in large part by a fear of poor performance. This brings into consideration the extent to which there exist student profiles based on different combinations of LRI and psychological challenge and threat orientations. LPA is designed to explore such possibilities and thus represents an important (and novel) means of assessing the study's contended network of instructional-psychological profiles (i.e., the within-network validity aspect of the study).

Consistent with Martin et al. (2021), recent challenge-threat frameworks (e.g., Putwain and Symes, 2014, 2016; Putwain et al., 2015; Uphill et al., 2019), and the latest approach-avoidance perspectives that have introduced growth goals (e.g., Elliot et al., 2011, 2015), we propose psychological challenge orientation can be inferred through students' self-efficacy and growth goals. Self-efficacy refers to a belief in one's capacity to meet or exceed a task demand or task challenge (Bandura, 1997; Schunk and DiBenedetto, 2014). This being the case, self-efficacy has been inferred as an analog of perceived challenge (Uphill et al., 2019) and operationalized as an indicator of perceived challenge in LPA (Martin et al., 2021). Perceived challenge has also been linked to approach orientations in motivational psychology. For example, Elliot defines approach motivation as the “energization of behavior by, or the direction of behavior toward, positive stimuli (objects, events, possibilities)” (2006, p. 112). According to Elliot, goals are a major means by which individuals' approach (and avoidance) orientations are manifested. Recent research has identified growth goals (i.e., self-improvement, or personal best goals) as one example of an approach motivation orientation (Martin and Liem, 2010; Elliot et al., 2011, 2015; Martin and Elliot, 2016a,b; Burns et al., 2018, 2019, 2020b). We recognize mastery goals are also approach-oriented, but it has previously been argued that growth goals represent a particularly ambitious and challenge-oriented goal striving (in keeping with our intent to capture challenge orientation) and are shown to explain variance in engagement beyond the effects of mastery goals (Yu and Martin, 2014; Martin and Elliot, 2016a). Growth goals are thus inferred as having an underlying psychological challenge dimension to them, along the lines of Uphill et al.'s (2019) review.

Also following Martin et al. (2021) and recent challenge-threat and approach-avoidance frameworks (e.g., Elliot et al., 2011, 2015; Putwain and Symes, 2014, 2016; Putwain et al., 2015; Uphill et al., 2019), we propose psychological threat orientation can be inferred through students' anxiety and failure avoidance goals. Anxiety reflects an individual's perception that a task demand exceeds their personal resources and capacity and poses a self-relevant threat to them in some way (Britton et al., 2011). Anxiety has thus been associated with threat appraisals (e.g., see Putwain et al., 2015, 2017; see also Uphill et al., 2019) and has been used as an indicator of perceived threat in LPA (Martin et al., 2021). Perceived threat has also been linked to avoidance orientations in motivational psychology. Elliot defines avoidance motivation as the “energization of behavior by, or the direction of behavior away from, negative stimuli (objects, events, possibilities)” (2006, p. 112). In goal theory research, avoidance goals are a salient example of avoidance motivation (Elliot, 2006; Van Yperen et al., 2015). Avoidance goals are those where the individual strives to avoid poor performance and failure or the implications of poor performance (Covington, 2000; Elliot, 2006; Martin, 2007, 2009), and being an element of an avoidance orientation, are suggested as analogs of an inherent psychological threat orientation, along the lines of Uphill et al.'s (2019) review.

To summarize, the present study assesses students' self-efficacy and growth goals (to infer challenge) and anxiety and failure avoidance goals (to infer threat) as key psychological orientations to include as indicators alongside LRI in LPA. Importantly, however, although we conceptually categorize these as challenge and threat indicators, they are modeled as separate indicators so that these conceptual groupings can be tested empirically. We also point out that this study is a multilevel one that assesses these issues at the classroom-level. It is feasible that psychological orientations of challenge and threat manifest at the classroom-level such that some classrooms are relatively higher or lower in these orientations. Indeed, motivation research has suggested that classrooms can vary in the extent to which they are challenge- and approach-oriented *vs*. threat- and avoidance-oriented (Lau and Nie, 2008). Furthermore, these classroom-level psychological orientations may co-vary with different levels of LRI in different ways. This being the case, the present study assesses classroom-level psychological orientations alongside classroom-level LRI to identify classroom-level instructional-psychological profiles.

## Assessing Links Between Instructional-Psychological Profiles and Academic Outcomes

This research is conducted in the educational domain of science. Science is a challenging subject for many students (Coe et al., 2008) and there are worrying trends in students' science achievement and science participation (especially in “Western” nations). For example, in the Programme for International Student Assessment (PISA), the long-term change in the average science performance of Australia (the site of the present study) demonstrates one of the largest declines among participating nations (OECD., 2020). There are also long-term declines in science participation and enrolments among senior school students (Office of the Chief Scientist, 2014) as well as declining interest in science in high school (Tröbst et al., 2016). Inherent in these trends are three major concerns. First, students do not seem to be orienting toward science in a participatory and persistent way—Martin et al. (2012) referred to this as “switching on.” Second, there are unacceptable numbers of students disengaging from science—Martin et al. (2012) referred to this phenomenon as “switching off.” Third, science achievement is in decline in many nations. This being the case, it is important to: (a) initiate and foster more positive persistence (“switching on”) in science, (b) arrest students' disengagement (“switching off”) in science, and (c) boost students' science achievement. Therefore, we sought to explore the association between the derived network of student- and classroom-level instructional-psychological profiles and a network of student- and classroom-level outcomes in the form of science persistence, disengagement, and achievement. From a construct validation perspective (Marsh, 2002), this represents the between-network validity aspect of our investigation.

In variable-centered research, Martin et al. (2012) found that approach-oriented predictors such as self-efficacy were positively associated with “switching on” in mathematics (positive future intentions and engagement) and negatively associated with “switching off” in mathematics (disengagement). The inverse pattern of associations was found for avoidance-oriented predictors such as anxiety. Also, in variable-centered research, LRI is positively associated with achievement in mathematics (Martin and Evans, 2018). In recent person-centered research, Martin et al. (2021) found that approach (challenge)-oriented students had higher science test scores, while avoidance (threat)-oriented students had lower scores. Interestingly, and in keeping with the potential dual presence of challenge and threat (Uphill et al., 2019), Martin et al. (2021) also identified a third profile reflecting both approach (challenge) and avoidance (threat)—students in this profile scored midway between the former two profiles on the science test. Taking these recent student-level findings together, we tentatively suggest at least three student-level profiles that will be associated with our outcomes (persistence, disengagement, achievement) in a descending adaptive pattern: high approach-low avoidance-high LRI, high approach-high avoidance-high LRI, and low approach-high avoidance-low LRI. From a construct validity perspective, demonstrating associations in a predicted manner is important (Marsh et al., 2006). Regarding classroom-level findings, we believe there is not a sufficient evidence base to guide predictions and so this is a more exploratory aspect of the study.

## Aims of the Present Study

We argue that to fully understand instructional cognitive load, it is important to operationalize and assess it in novel ways that can provide unique validity insights into how different students perceive and experience this load. We further suggest it is important to consider these novel assessment approaches using appropriate cutting-edge analytic models. Accordingly, we adopted a within- and between-network construct validity approach and used multilevel LPA to identify instructional-psychological profiles among students and classrooms based on students' reports of instructional cognitive load (via load reduction instruction; LRI) and their accompanying psychological challenge orientations (self-efficacy, growth goals) and psychological threat orientations (anxiety, failure avoidance goals). In phase 1 of analyses, we sought to identify a network of instructional-psychological profiles among students (student-level within-network validity). In phase 1, we also tested the links between the derived profile network and a network of three academic outcomes (persistence, disengagement, achievement) (student-level between-network validity). In phase 2 of analysis, we extended our examination to the classroom-level where we sought to identify the network of classroom profiles based on the relative frequency of student profiles identified in phase 1 (classroom-level within-network validity). We also tested whether the derived network of different classroom profiles was associated with different levels of classroom-average persistence, disengagement, and achievement (classroom-level between-network validity). Figure 1 demonstrates the model and parameters under investigation.

## Method

### Participants and Procedure

Participants comprised 2,071^1^ Australian high school students from 188 science classrooms in eight schools. The schools were independent (non-systemic) schools, in a major capital city on the east coast of Australia. Four of these schools were single-sex boys' schools and four were single-sex girls' schools. Just over half (60%) the sample comprised girls. Participants were in Year 7 (29%), Year 8 (22%), Year 9 (24%), and Year 10 (25%). The average age was 14.02 years (*SD* = 1.27 years). Just under one in ten (8%) students spoke a language other than English at home. Socioeconomic status (SES) varied (range 846–1,181, *M* = 1,138, *SD* = 41, on the Australian Bureau of Statistics Index of Relative Socio-Economic Advantage and Disadvantage classification), but in aggregate was a higher SES than the Australian mean of 1,000. On average, classrooms had about eleven students (adequate for group-level effect estimation and not disproportionate to the ratio of staff to students in the independent school sector when accounting for student absences, non-participation, and students who have not received participation consent from their parents; also see Australian Bureau of Statistics, 2019). The lead researcher's university provided human ethics approval for the study. Approval was then provided by school principals for their school to participate. Then, participating students (and their parents/carers) provided consent. The online survey (that also comprised an achievement test) was administered during a regularly scheduled science class in 2018. Students completed the instrument on their own. Teachers were informed they could help students with procedural aspects of the survey, but not provide assistance in answering specific questions. The data in this investigation are shared with Martin et al. (2020a), who conducted a variable-centered study exploring the extent to which class participation, educational aspirations, and enjoyment of school mediated the relationship between LRI and achievement.

### Materials

Indicators for the network of instructional-psychological profiles were measured by way of instructional cognitive load (load reduction instruction), psychological challenge orientation (self-efficacy, growth goals), and psychological threat orientation (anxiety, failure avoidance goals). These indicators were the within-network validity variables for this study. The network of outcome measures was assessed by way of persistence, disengagement, and achievement. These were the between-network validity variables for the study.

#### Instructional Cognitive Load via Load Reduction Instruction Scale—Short (LRIS-S)

As described in Martin et al. (2020a), the LRIS-S was developed to measure student perceptions of their teacher's use of instructional strategies known to reduce extraneous cognitive load (and because of this, some intrinsic cognitive load). In the LRIS-S, the five LRI factors are represented by a single item (the full LRIS has 5 items for each of the 5 factors; Martin and Evans, 2018). As detailed in Martin et al. (2020a), the factors and items (adapted to science) are: *difficulty reduction* (“When we learn new things in this science class, the teacher makes it easy at first”); *support* (“In this science class, the teacher is available for help when we need it”); *practice* (“In this science class, the teacher makes sure we practice important things we learn”); *feedback-feedforward* (“In this science class, the teacher provides frequent feedback that helps us learn”); and *independence* (“Once we know what we're doing in this science class, the teacher gives us a chance to work independently”). Responses were provided on a seven-point scale (1 = *strongly disagree* to 7 = *strongly agree*). Reliability for this scale was sound (see Table 1) and ICC = 0.16. Because the LRIS has an emphasis on reduction of cognitive load, Martin and Evans (2018) conducted analyses showing that the scale was significantly negatively associated with intrinsic load (load referring to task difficulty and complexity) and extraneous load (load referring to difficulty and complexity of instruction; Chandler and Sweller, 1991). They concluded that the LRIS does assess aspects of instruction impacting distinct elements of cognitive load. Martin et al. (2020a) showed that the reliability of the LRIS-S at student- and classroom-levels was high and their doubly-latent multi-level (student and classroom) factor analysis demonstrated sound fit and yielded strong factor loadings. Thus, we suggest that student-reported LRIS-S offers valid insights into the instructional practices relevant to cognitive load.

**Table 1:** Descriptive statistics.

	**Student-level (Level 1; L1)**			**Classroom-level (Level 2; L2)**		
	***M***	***SD***	**ω–Coefficient omega**	***M***	***SD***	**ω–Coefficient omega**
**Profile Network Indicators**						
LRI	5.284	1.123	0.85	5.298	0.626	0.96
Self-efficacy	5.790	1.025	0.83	5.778	0.529	0.93
Growth Goals	5.202	1.152	0.90	5.200	0.655	0.99
Anxiety	4.113	1.362	0.78	4.168	0.650	0.94
Failure Avoid Goals	3.194	1.396	0.83	3.250	0.665	0.98
**Outcome Network**						
Persistence	5.146	1.105	0.83	5.137	0.614	0.97
Disengagement	2.387	1.332	0.87	2.401	0.765	0.98
Achievement	0.000	1.000	0.79	0.000	1.000	0.86

*Achievement is standardized; LRI, load reduction instruction*.

#### Psychological Challenge Orientation

Psychological challenge orientation was assessed via self-efficacy and growth goals. Self-efficacy (4 items; e.g., “If I try hard, I believe I can do well in this science class”) was measured via the domain-specific form of the Motivation and Engagement Scale—High School (MES-HS; Martin, 2007, 2009), validated by Green et al. (2007). Growth goals (4 items; e.g., “When I do my science schoolwork I try to do it better than I've done before”) were measured via the domain-specific form of the Personal Best Goal Scale (Martin and Liem, 2010), for which Martin and Elliot (2016a) provided evidence of validity. Responses were provided on a seven-point scale (1 = *strongly disagree* to 7 = *strongly agree*). Reliability for the scores of both scales was sound (see Table 1) and ICCs = 0.08 and 0.12 for self-efficacy and growth goals, respectively.

#### Psychological Threat Orientation

Psychological threat orientation was assessed via anxiety (4 items; e.g., “When exams and assignments are coming up for this science class, I worry a lot”) and failure avoidance goals (4 items; e.g., “Often the main reason I work in this science class is because I don't want people to think that I'm dumb”). Both were from the domain-specific form of the MES-HS (Martin, 2007, 2009), for which Green et al. (2007) provided evidence of validity. Responses were provided on a seven-point scale (1 = *strongly disagree* to 7 = *strongly agree*). Reliability for the scores of both scales was sound (see Table 1) and ICCs = 0.08 and 0.05 for anxiety and failure avoidance goals, respectively.

#### Persistence and Disengagement

In line with Martin et al. (2012), engagement was assessed via the dual dimensions of “switching on” and “switching off.” Switching on was operationalized through persistence (4 items; e.g., “If I can't understand something in this science class at first, I keep going over it until I do”). Switching off was operationalized through disengagement (4 items; e.g., “Each week I'm trying less and less in this science class”). Both were from the domain-specific form of the MES-HS (Martin, 2007, 2009), validated by Green et al. (2007). Responses were provided on a seven-point scale (1 = *strongly disagree* to 7 = *strongly agree*). Reliability for scores of both scales was sound (see Table 1) and ICCs = 0.12 and 0.17 for persistence and disengagement, respectively.

#### Achievement

Achievement was measured using 12 questions in an online test (part of the online survey). Instrument piloting and development are fully described in Martin et al. (2020a). The test aligned with the science syllabus applicable to our sample; therefore, two forms were developed, one based on the Stage 4 (years 7 and 8) state science syllabus and the other based on the Stage 5 (years 9 and 10) state science syllabus. Test questions were set within the contexts of Physical World, Earth and Space, Living World, and Chemical World and addressed the following skills: questioning and predicting, planning investigations, conducting investigations, processing and analyzing data and information, and problem solving. Each question was grounded within one of the abovementioned specific science contexts and there was an ~30/70 ratio of content-focused to skill-focused questions, with the easier questions focusing on content and the harder questions focusing on skill application. All multiple-choice test responses were recoded as dichotomous (0 = incorrect; 1 = correct). The correct answers were summed to a total achievement score (thus, a continuous scale), reflecting something of a formative construct. Achievement scores were then standardized by year level (*M* = 0; *SD* = 1). The test was reliable, as shown in Table 1 and ICC = 0.37.

## Data Analysis

Analyses were conducted using M*plus* 8.60 (Muthén and Muthén, 2017). The robust maximum likelihood (MLR) estimator was used in all models. Missing data were addressed using Full Information Maximum Likelihood (FIML; Arbuckle, 1996). Confirmatory factor analysis (CFA) was run at the student-level (and corrected for nesting within classrooms via the M*plus* “COMPLEX” command) using the standardized factor approach to identification to obtain student-level factor scores for the five profile indicators and the three outcomes. The CFA also comprised background attributes as auxiliary variables—reported in analyses in Supplementary Materials. Factor scores were saved and used in the LPAs. The LPA analyses comprised two phases: single-level LPA (phase 1) and multi-level LPA (phase 2).

### Single-Level LPA

For the single-level LPA conducted at the student-level (Level 1; L1), we tested a range of solutions involving 1 through 9 profiles. Following Collie et al. (2020), variances and means were free to differ across profiles and indicator variables; models were estimated using at least 10,000 random start values, with 100 iterations and 1,000 final stage optimizations; and we confirmed that the best log-likelihood value was replicated for each model. Numerous indices were used to assess model fit: for the Consistent Akaike Information Criteria (CAIC), Akaike Information Criteria (AIC), Bayesian Information Criteria (BIC), and sample-size-adjusted Bayesian Information Criteria (SSA-BIC), smaller values reflect better fit. We created elbow plots of the CAIC, AIC, BIC, and SSA-BIC indices. In these plots, the profile at which point the slope noticeably flattens is another indicator of an appropriate solution (Morin et al., 2016). The *p*-value of the adjusted Lo–Mendell–Rubin Likelihood Ratio Test (*p*LMR) enabled comparison of a *k*-profile model against a *k*-1 profile model to determine if the former profile model yielded a better fit relative to the latter profile model. We also provide entropy values in Table 2 as an indicator of classification accuracy. In addition to fit indices and where appropriate, we applied rules of parsimony, conceptual relevance, and statistical adequacy to further ascertain the optimal solution. After identifying the final network of profiles (the student-level within-network validity aspect), we then examined the network of academic outcomes (persistence, disengagement, achievement) as a function of profile membership (the student-level between-network validity aspect), controlling for background attributes. Outcomes were included using the direct approach and compared across profiles using the M*plus* “MODEL CONSTRAINT” option, which relies on the multivariate delta method for tests of statistical significance (e.g., Raykov and Marcoulides, 2004). As part of this, the outcomes were also regressed on participants' background attributes, which acted as covariate controls (McLarnon and O'Neill, 2018).

**Table 2:** Single-level LPA fit statistics.

	**1 Profile**	**2 Profiles**	**3 Profiles**	**4 Profiles**	**5 Profiles**	**6 Profiles**	**7 Profiles**	**8 Profiles**	**9 Profiles**
*N*	2,071	2,071	2,071	2,071	2,071	2,071	2,071	2,071	2,071
Free Parameters	10	21	32	43	54	65	76	87	98
Log-likelihood	−13,965	−12,628	−12,169	−11,871	−11,628	−11,439	−11,309	−11,167	−11,078
CAIC	28,016	25,437	24,614	24,113	23,722	23,439	23,274	23,085	23,002
Akaike (AIC)	27,951	25,298	24,402	23,828	23,365	23,008	22,771	22,509	22,353
Bayesian (BIC)	28,007	25,417	24,582	24,070	23,669	23,375	23,199	22,999	22,905
S-SA BIC	27,975	25,350	24,480	23,934	23,498	23,168	22,958	22,723	22,593
Entropy	–	0.782	0.844	0.792	0.782	0.803	0.815	0.815	0.793
*p*LMR	–	<0.001	<0.001	<0.001	<0.001	0.002	0.041	0.521	0.341

*N, sample size; AIC, Akaike Information Criteria; CAIC, Consistent Akaike Information Criteria; S-SA, Sample-Size Adjusted; BIC, Bayesian Information Criteria; pLMR, Lo–Mendell–Rubin Likelihood Ratio Test*.

### Multi-Level LPA

In phase 2, we extended the student-level (Level 1, L1) findings to determine the extent to which classroom-level (Level 2; L2) profiles could be identified; or, put another way, the extent to which we could identify classroom profiles characterized by distinct combinations of the different student profiles. Thus, phase 2 identified classroom profiles based on the relative frequency of the various L1 latent profiles. To maintain the stability of the previously identified student-level profiles (L1), we used the manual 3-step approach detailed by Litalien et al. (2019; also see Vermunt, 2010; Morin and Litalien, 2017). Multi-level LPA solutions (1–9 classroom-level profiles) were assessed. Following Collie et al. (2020), each model was estimated using at least 10,000 random start values, 100 iterations, and 1,000 final stage optimizations; replication of the best log-likelihood value was sought for each model; and the best model was selected using the same criteria as the single-level LPA (phase 1), except the *p*LMR, which is not available for multi-level LPA. After identifying the network of classroom-level profiles (the classroom-level within-network validity aspect), we then examined the network of L2 outcomes (classroom-average persistence, disengagement, and achievement) as a function of profile membership by adding classroom-average outcome variables (using the M*plus* cluster mean approach) to the final best-fitting model (the classroom-level between-network validity aspect). Outcomes were compared across profiles using the M*plus* “MODEL CONSTRAINT” option (e.g., Raykov and Marcoulides, 2004).

## Results

### Preliminary Analyses

Table 1 shows reliability coefficients and descriptive statistics for the profile indicators and the outcome variables in the study. These data indicate acceptable reliability. The CFA used to generate factor scores yielded an excellent fit to the data, χ^2^ = 1,321, df = 378, *p* < 0.001, CFI = 0.964, TLI = 0.958, RMSEA = 0.035. Indeed, these preliminary variable-centered analyses demonstrate sound within-network validity properties (Marsh, 2002). The resulting correlation matrix is presented in Supplementary Table 1. These factor scores were then used in the subsequent LPAs.

### Single-Level LPA

**Figure 2:**
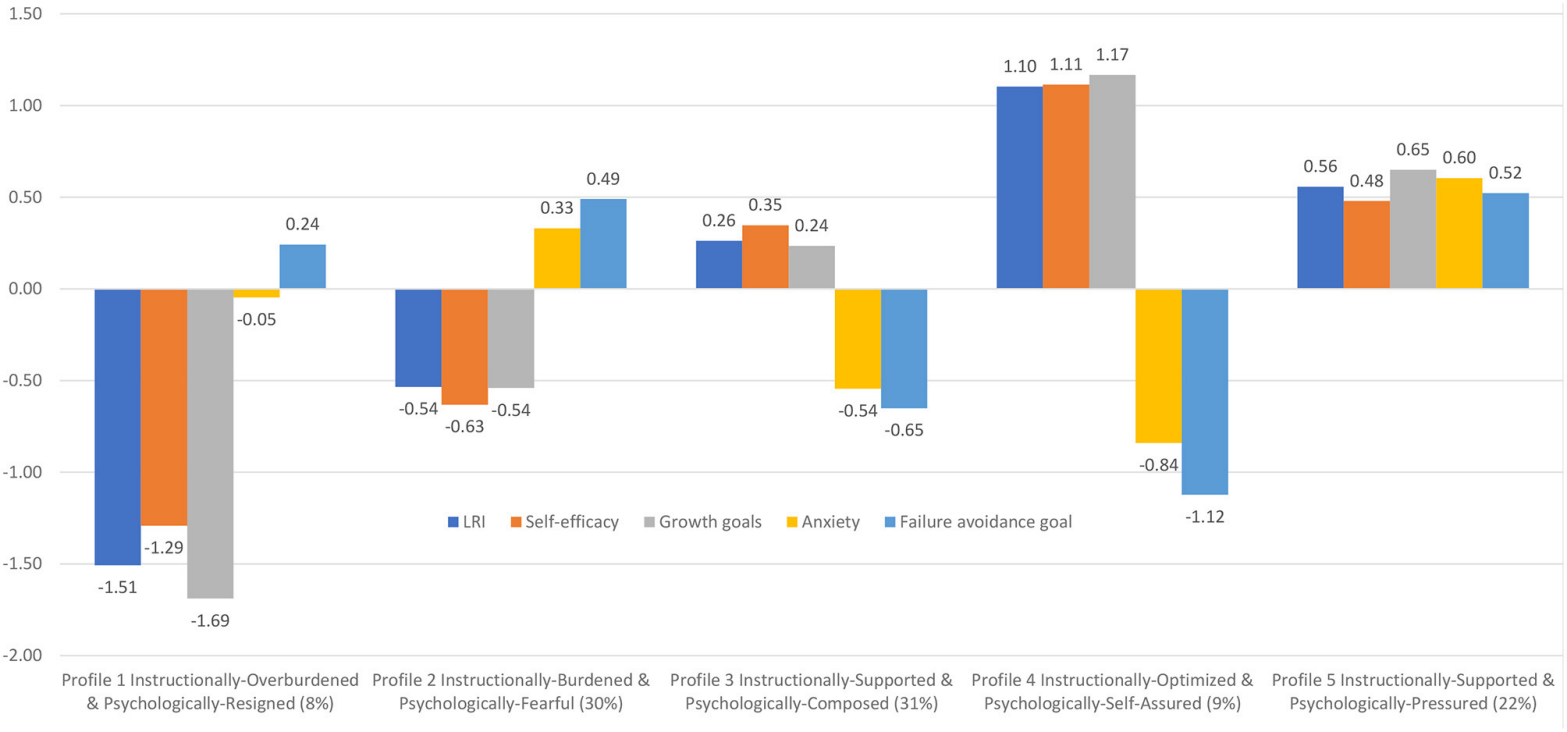
Single-level LPA results: Instructional-psychological profile names, profile means, and % of sample.

The fit statistics for the 1–9-profile solutions are shown in Table 2 and the elbow plot is shown in Supplementary Figure 1. In these it is evident that the CAIC, AIC, BIC, and SSA-BIC decline with each additional profile. There appears to be slight inflection points around 4, 5, and 6 profiles. Although we do not rely on the *p*LMR, it is interesting to note it supported the 6-profile solution, but it was significant at the *p* < 0.01 level—whereas the 4–5-profile solutions were significant at *p* < 0.001. In addition, although not relying on minimum profile size as a decision criterion, we note that the 6-profile solution had a minimum profile size of <2%, whereas the 5-profile solution had a size of 8%. Taken together, we felt that additional profiles were theoretically useful and well-differentiated up to 6 profiles, but the sixth profile presented a shape that was qualitatively similar (even if it differed quantitatively) to that of the 5-profile solution. We therefore proceeded with the 5-profile solution. A graphical representation of this 5-profile solution is presented in Figure 2. Students corresponding to profile 1 (8% of students) reported very low LRI, very low self-efficacy, very low growth goals, neutral anxiety, and neutral failure avoidance goals. This profile was labeled Instructionally-Overburdened & Psychologically-Resigned to reflect very high instructional cognitive load and very low challenge orientation, indeed so much so they are not particularly threatened, but rather resigned. Students corresponding to profile 2 (30% of students) reported low LRI, low self-efficacy, low growth goals, high anxiety, and high failure avoidance goals. This profile was labeled Instructionally-Burdened & Psychologically-Fearful to reflect modest instructional load, low challenge orientation, and high threat orientation. Students corresponding to profile 3 (31% of students) reported above average LRI, above average self-efficacy and growth goals, and below average anxiety and failure avoidance goals. We labeled this profile Instructionally-Supported & Psychologically-Composed to reflect low instructional load, above average challenge orientation, and below average threat orientation. Students corresponding to profile 4 (9% of students) reported very high LRI, very high self-efficacy, very high growth goals, very low anxiety, and very low failure avoidance goals. We labeled this profile Instructionally-Optimized & Psychologically-Self-Assured to reflect the very low cognitive instructional load, the very high challenge orientation, and the very low threat orientation. Students corresponding to profile 5 (22% of students) reported above average scores on each of LRI, self-efficacy, growth goals, anxiety, and failure avoidance goals. We labeled this profile Instructionally-Supported & Psychologically-Pressured to reflect low instructional load as well as dual high challenge and threat orientations.

We then assessed for differences between profiles in persistence, disengagement, and achievement (adjusted for background attribute covariates—see Supplementary Tables 2A–E for the predictive relationships between background attributes and the latent profiles). Mean scores are shown in Table 3. For persistence, findings indicated that each profile was significantly different from the other. In ascending order of persistence were: Instructionally-Overburdened & Psychologically-Resigned (lowest persistence; *M* = −1.571), then Instructionally-Burdened & Psychologically-Fearful (*M* = −0.375), then Instructionally-Supported & Psychologically-Composed (*M* = 0.559), then Instructionally-Supported & Psychologically-Pressured (*M* = 0.857), then Instructionally-Optimized & Psychologically-Self-Assured (highest persistence; *M* = 1.407).

**Table 3:** Means (SEs and 95% CIs) on dependent variables for each instructional-psychological profile.

	**Persistence**				**Disengagement**				**Achievement**			
	**Mean**	**SE**	**95% CI**		**Mean**	**SE**	**95% CI**		**Mean**	**SE**	**95% CI**	
Instructionally-Overburdened & Psychologically-Resigned	−1.571^a^	0.097	−1.762	−1.381	1.660^a^	0.088	1.487	1.834	−0.590^a^	0.146	−0.876	−0.304
Instructionally-Burdened & Psychologically-Fearful	−0.375^b^	0.076	−0.524	−0.227	0.436^b^	0.056	0.328	0.545	−0.409^a^	0.118	−0.640	−0.179
Instructionally-Supported & Psychologically-Composed	0.559^c^	0.074	0.414	0.704	−0.683^c^	0.060	−0.800	−0.567	0.089^b^	0.115	−0.136	0.313
Instructionally-Optimized & Psychologically-Self-Assured	1.407^d^	0.072	1.222	1.549	−1.231^d^	0.040	−1.335	−1.152	0.430^c^	0.119	0.123	0.664
Instructionally-Supported & Psychologically-Pressured	0.857^e^	0.074	0.711	1.003	−0.743^c^	0.061	−0.862	−0.625	0.054^d^	0.116	−0.174	0.281

*Different superscripts in a given column indicate a significant difference between means at p < 0.05; SE, standard error; CI, confidence interval*.

For disengagement, findings indicated that with one exception (Instructionally-Supported & Psychologically-Composed = Instructionally-Supported & Psychologically-Pressured), each profile was significantly different from the other. In descending order of disengagement were: Instructionally-Overburdened & Psychologically-Resigned (highest disengagement; *M* = 1.660), then Instructionally-Burdened & Psychologically-Fearful (*M* = 0.436), then Instructionally-Supported & Psychologically-Composed and also Instructionally-Supported & Psychologically-Pressured (*M* = −0.683 and *M* = −0.743, respectively), then Instructionally-Optimized & Psychologically-Self-Assured (lowest disengagement; *M* = −1.231).

For achievement, findings indicated that with one exception (Instructionally-Overburdened & Psychologically-Resigned = Instructionally-Burdened & Psychologically-Fearful), each profile was significantly different from the other. In ascending order of achievement were: Instructionally-Overburdened & Psychologically-Resigned and also Instructionally-Burdened & Psychologically-Fearful (lowest achievement; *M* = −0.590 and *M* = −0.409, respectively), then Instructionally-Supported & Psychologically-Pressured (*M* = 0.054), then Instructionally-Supported & Psychologically-Composed (*M* = 0.089), then Instructionally-Optimized & Psychologically-Self-Assured (highest achievement; *M* = 0.430).

### Multi-Level LPA

The fit statistics for the multi-level LPA solutions are reported in Table 4 (the elbow plot is shown in Supplementary Figure 2). Here 1–9-profile solutions are presented. The 2-profile solution resulted in the consistently lowest value for the fit indices, but there was some further flattening on other indices at the third profile. Also, as described below, the 3-profile solution yielded a group that separated classrooms in qualitatively distinct ways that was beyond what was possible in a 2-profile solution that (as it turned out, and is described below) could not differentiate a Striving profile, from Thriving and Struggling profiles. Moreover, this additional profile constituted a sizeable subpopulation (22%). Morin et al. (2017) emphasize the importance of ensuring that each profile adds conceptually and practically meaningful information to a solution. Thus, while recognizing aspects of fit suggest a 2-profile solution, we concluded there was substantive and practical yield in the additional profile. Accordingly, a solution with 3 classroom-level profiles was selected as the final solution.

**Table 4:** Multi-level LPA fit statistics.

	**1 Profile**	**2 Profiles**	**3 Profiles**	**4 Profiles**	**5 Profiles**	**6 Profiles**	**7 Profiles**	**8 Profiles**	**9 Profiles**
*N*	2,071	2,071	2,071	2,071	2,071	2,071	2,071	2,071	2,071
Free Parameters	4	9	14	19	24	29	34	39	44
Log-likelihood	−3,061	−3,003	−2,994	−2,989	−2,987	−2,982	−2,981	−2,981	−2,982
CAIC	6,157	6,084	6,109	6,142	6,181	6,214	6,256	6,299	6,344
Akaike (AIC)	6,131	6,025	6,016	6,017	6,023	6,022	6,030	6,041	6,053
Bayesian (BIC)	6,153	6,075	6,095	6,124	6,158	6,185	6,222	6,261	6,301
S-SA BIC	6,141	6,047	6,050	6,064	6,082	6,093	6,114	6,137	6,161
Entropy	0.669	0.664	0.687	0.655	0.617	0.633	0.607	0.662	0.649

*N, sample size; AIC, Akaike Information Criteria; CAIC, Consistent Akaike Information Criteria; S-SA, Sample-Size Adjusted; BIC, Bayesian Information Criteria*.

**Figure 3:**
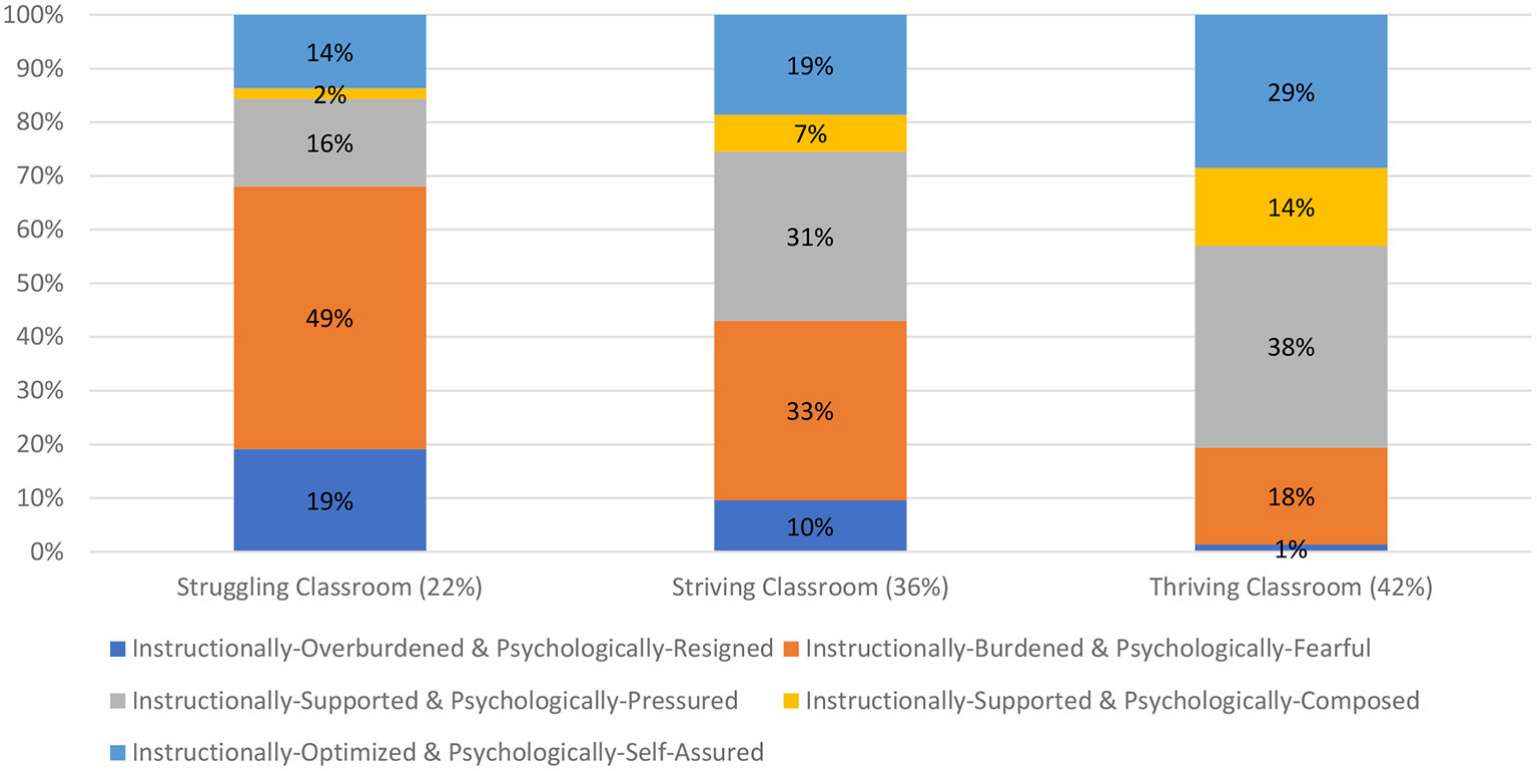
Multi-level LPA results showing the classroom-level instructional-psychological profiles.

A graphical representation of this final 3-profile solution is presented in Figure 3. Examination of this 3-profile solution suggested the presence of one Struggling classroom profile (22% of the classrooms), one Striving classroom profile (36% of the classrooms), and one Thriving classroom (42% of classrooms). The Struggling classroom had the highest proportion of students from the Instructionally-Overburdened & Psychologically-Resigned (19%) and Instructionally-Burdened & Psychologically-Fearful (49%) profiles. The Striving classroom included a high proportion of students from the Instructionally-Supported & Psychologically-Pressured (31%), Instructionally-Burdened & Psychologically-Fearful (33%), and Instructionally-Overburdened & Psychologically-Resigned (10%) profiles. The Thriving classroom had the highest proportion of students from the Instructionally-Optimized & Psychologically-Self-Assured (29%) profile, along with a high proportion of students from the Instructionally-Supported & Psychologically-Composed (14%) and Instructionally-Supported & Psychologically-Pressured (38%) profiles.

We then assessed for differences between classroom profiles in classroom-average persistence, disengagement, and achievement. Results are shown in Table 5. For classroom-average persistence, each classroom profile was significantly different from the other. In ascending order of classroom-average persistence were: the Struggling classroom (lowest persistence; *M* = −0.610), then the Striving classroom (*M* = −0.068), then the Thriving classroom (highest persistence; *M* = 0.422). For classroom-average disengagement, each classroom profile was significantly different from the other. In descending order of classroom-average disengagement were: the Struggling classroom (highest disengagement; *M* = 0.705), then the Striving classroom (*M* = 0.048), then the Thriving classroom (lowest disengagement; *M* = −0.427). For classroom-average achievement, with one exception (Striving = Thriving), each classroom profile was significantly different from the other. In ascending order of classroom-average achievement were: the Struggling classroom (lowest achievement; *M* = −0.498), then the Striving classroom (*M* = −0.032), then the Thriving classroom (highest achievement; *M* = 0.182)—but as noted, the latter two classroom profiles were not significantly different from each other in achievement (see Table 5).

**Table 5:** Means (and SEs and 95% CIs) on dependent variables for each instructional-psychological profile.

	**Persistence**				**Disengagement**				**Achievement**			
	**Mean**	**SE**	**95% CI**		**Mean**	**SE**	**95% CI**		**Mean**	**SE**	**95% CI**	
Struggling Classrooms	−0.610^a^	0.085	−0.776	−0.443	0.705^a^	0.101	0.506	0.903	−0.498^a^	0.067	−0.630	−0.367
Thriving Classrooms	0.422^b^	0.048	0.329	0.515	−0.427^b^	0.037	−0.500	−0.354	0.182^b^	0.072	0.042	0.322
Striving Classrooms	−0.068^c^	0.074	−0.213	0.077	0.048^c^	0.086	−0.120	0.215	−0.032^b^	0.115	−0.257	0.194

*Different superscripts in a given column indicate a significant difference between means at p < 0.05; SE, standard error; CI, confidence interval*.

## Discussion

To best understand instructional cognitive load, we have emphasized the importance of assessing it in novel ways to reveal how different students perceive and experience this load. We have further emphasized the importance of utilizing cutting-edge analytic approaches that are appropriate to assessing these novel instrumentations. Integrating cognitive load theory and cognitive appraisal theory, we hypothesized that some students are likely to perceive cognitive load in an approach- and challenge-oriented way, and other students are likely to perceive cognitive load in an avoidant- and threat-oriented way. To the extent this is the case, we suggested that to further understand instructional cognitive load (by way of load reduction instruction; LRI) it is important to do so by also assessing students' accompanying psychological challenge and threat orientations. Adopting a novel person-centered construct validity perspective, we used latent profile analysis (LPA) to identify the network of instructional-psychological profiles based on students' reports of instructional load (LRI) and their accompanying psychological challenge orientations (self-efficacy and growth goals) and psychological threat orientations (anxiety and failure avoidance goals)—student-level within-network validity. Moreover, because students in our study were nested within (science) classrooms, we expanded our analyses to also conduct multilevel LPA to identify a network of student- and classroom-level instructional-psychological profiles—classroom-level within-network validity. We assessed student- and classroom-level between-network validity by investigating associations between the network of derived profiles and the network of student- and classroom-level persistence, disengagement, and achievement outcome variables.

### Summary of Findings

At the student-level, we identified five instructional-motivational profiles that represented different presentations of instructional cognitive load, challenge orientation, and threat orientation: Instructionally-Overburdened & Psychologically-Resigned students (8% of students), Instructionally-Burdened & Psychologically-Fearful students (30%), Instructionally-Supported & Psychologically-Composed students (31%), Instructionally-Optimized & Psychologically-Self-Assured students (9%), and Instructionally-Supported & Psychologically-Pressured students (22%). As we describe below, these conform to established theoretical perspectives and thus offer a student-level within-network validation perspective on the nomological network of instructional cognitive load in terms of underlying instructional-psychological orientations. We also demonstrated student-level between-network validity in that these profiles were significantly different in persistence, disengagement, and achievement (beyond the role of background attributes)—with the Instructionally-Overburdened & Psychologically-Resigned profile reflecting the most maladaptive outcomes and the Instructionally-Optimized & Psychologically-Self-Assured profile reflecting the most adaptive outcomes. In multilevel LPAs, we identified three instructional-psychological profiles among classrooms that varied in terms of instructional cognitive load, challenge orientations, and threat orientations: Striving classrooms (36% of the classrooms), Thriving classrooms (42%), and Struggling classrooms (22%). In terms of classroom-level between-network validity, we found that classroom profiles were significantly different in their levels of persistence, disengagement, and achievement—with Struggling classrooms reflecting the most maladaptive outcomes and Thriving classrooms reflecting the most adaptive outcomes, but, notably, equal to the Striving classrooms in achievement.

### Findings of Particular Note

In numerous ways this study offers novel contributions to the assessment of instructional cognitive load, including: its person-centered perspective elucidating theoretically plausible student profiles based on their experience of cognitive load and their psychological orientations, the multilevel validity of the Load Reduction Instruction Scale-Short (LRIS-S) in person-centered analyses, and the validity of the links between profiles and academic outcomes. We suggest that findings hold implications for better assessing and understanding students and classrooms in terms of the cognitive load they experience through instruction. Specifically, the results show that assessing instructional load in the context of students' accompanying psychological orientations can reveal unique insights about students' learning experiences and about important differences between classrooms in terms of the instructional load that is present.

The findings supported one of the central premises of this study—namely, that similar levels of perceived instructional load can be accompanied by different levels of perceived challenge and threat. For example, at the student-level we identified two profiles that can be considered instructionally-supported but who varied in their accompanying psychological orientations. Specifically, the Instructionally-Supported & Psychologically-Composed profile experienced moderate levels of LRI, moderate challenge orientation and low threat orientation, whereas the Instructionally-Supported & Psychologically-Pressured profile experienced moderate LRI and challenge orientation but also moderate levels of threat orientation. These two profiles were also significantly different in persistence and achievement outcomes (but not disengagement), with the former profile scoring higher than the latter profile. This is notable because it shows that students with similar levels of instructional load can have different psychological experiences (i.e., differing levels of challenge and threat) that yield significant differences in academic outcomes. This underscores the yield of assessing instructional load in the context of other potentially influential accompanying factors. This requires assessment and analytic approaches that can disentangle students who perceive similar levels of instructional load but who vary on other factors (in our study, psychological challenge and threat orientations).

The Instructionally-Supported & Psychologically-Pressured profile was further illuminating in that it confirmed the existence of the contended dual challenge-threat orientation (or, approach-avoidance motive). As noted earlier, recent reviews of challenge-threat orientations have suggested the dual presence of both challenge and threat among some individuals (Uphill et al., 2019; see also Rogat and Linnenbrink-Garcia, 2019 for dual goals under approach-avoidance goal frameworks). In the case of our study, in the presence of instructional load there were some students who also reported dual challenge and threat orientations—that is, they believed they are up to the challenge of task burden but are also fearful of failure or poor performance, somewhat akin to the “overstrivers” described earlier (Covington, 2000; Martin and Marsh, 2003). Essentially, in the context of instructional load they perceive both an opportunity to succeed and a risk they may fail. Accordingly, we identified these students as Psychologically-Pressured because even though they reflected a challenge orientation, there was an accompanying fear and avoidance (threat) inclination. Moreover, despite their threat orientation, the presence of a concomitant challenge orientation meant they experienced higher academic outcomes relative to the Instructionally-Overburdened & Psychologically-Resigned students and the Instructionally-Burdened & Psychologically-Fearful students. Nonetheless, the dual presence of challenge-threat orientations experienced by the Psychologically-Pressured profile represented a tension that we contend held them back from the more optimal academic outcomes seen in the Psychologically-Composed and Psychologically-Self-Assured profiles; this aligns with recent research that similarly demonstrates that the benefits of challenge orientation can be thwarted when there are similarly high rates of threat (Burns et al., 2020a).

Another interesting finding was that the highest instructional cognitive load (i.e., the lowest LRI scores) was not accompanied by the highest levels of threat orientation. Specifically, the Instructionally-Overburdened & Psychologically-Resigned students reflected lower levels of anxiety and failure avoidance goals than the Instructionally-Burdened & Psychologically-Fearful students and the Instructionally-Supported & Psychologically-Pressured students. It seems that in conditions where the instructional load is most poorly managed (evidenced by the lowest LRI scores), students may abandon any investment in the lesson. According to self-worth theory (Covington, 2000), when students abandon motivationally aversive conditions there can be an alleviation of anxiety and fear as their competence and academic self-worth are no longer “on the line” and under threat. Importantly, however, as they abandon their investment in cognitively burdensome instruction, their academic outcomes also decline—as evidenced by their significantly lower levels of persistence and significantly higher levels of disengagement.

Interestingly, the Instructionally-Overburdened & Psychologically-Resigned students and the Instructionally-Burdened & Psychologically-Fearful students were not significantly different in achievement. Even though the latter profile did not experience such depths of burdensome instruction, this did not yield an achievement advantage for them. Here we again point out the importance of assessing accompanying challenge and threat orientations to understand potentially counter-intuitive effects of instruction on achievement: in this study, it unearthed the fact that Instructionally-Overburdened students were not significantly different in achievement than the Instructionally-Burdened students. The former profile was Psychologically-Resigned whereas the latter profile was Psychologically-Fearful. Again drawing on self-worth theory (Covington, 2000), when students abandon investment in a task demand there can be a concomitant alleviation of anxiety and fear (discussed above) that may mean their performance can be on a par with students who are still invested in the task demand but who are highly anxious and fearful. This is yet another example of how dually assessing instructional cognitive load and psychological orientations can help us better understand instructional effects—namely, assessing concomitant challenge and threat orientations has allowed us to understand why two profiles who differ in instructional load are similar in achievement.

Another novel contribution by this study involved the multilevel analyses that enabled us to identify distinct types of classrooms differentiated in terms of how they varied in instructional load (LRI) and accompanying challenge and threat orientations. Here we unearthed three classroom profiles: Struggling, Striving, and Thriving classrooms. The Struggling classrooms were predominated by a majority of students experiencing significant instructional cognitive (over)load and psychological detachment or fear. In contrast, the Thriving classrooms had almost no students who were cognitively (over)loaded and a majority of students with adaptive challenge orientations. These two classroom profiles may be considered somewhat predictable from a binary perspective, but the third classroom profile (the Striving classroom) was more nuanced and represents both cautionary and aspirational possibilities: cautionary in the sense that if not instructionally- and psychologically-supported, these Striving classrooms risk devolving to Struggling classrooms—but aspirational in that if they are better instructionally- and psychologically-supported, they can elevate to Thriving classrooms. Where the Striving and Thriving classrooms seemed to differ most was in the number of Psychologically-Self-Assured and Psychologically-Composed students (43% of Thriving classrooms; 26% of Striving classrooms)—the implication being that educators would do well to shift students “up” from the Psychologically-Pressured profile to the Psychologically-Self-Assured and Psychologically-Composed profiles. How they can do this is now the focus of discussion.

### Implications for Instructional Assessment, Evaluation, and Practice

The findings of this investigation hold implications for instructional assessment, evaluation, and practice. For instructional assessment and evaluation, the study has further demonstrated the validity of instrumentation that enables students to report on the extent to which instruction manages the cognitive load on them as they learn. The Load Reduction Instruction Scale (LRIS; and its brief form, Load Reduction Instruction Scale-Short, LRIS-S) is a student reporting tool that has been purposefully developed for in-class assessment of LRI. To date the LRI has been usefully employed in variable-centered research, and the present study has now revealed its utility in person-centered analyses. Furthermore, because the LRIS is completed in class, if enough classrooms are present in a study (as there were in our study), it can be used in multilevel analyses to gain a sense of LRI at the whole-class level. We therefore encourage the use of tools that enable in-class assessment of load-reducing instruction by students. Indeed, as Martin and Evans (2018) suggested, the LRIS may also be adapted to have teachers reflect on and attend to their own instructional practice.

Also on the matter of instructional assessment and evaluation, person-centered analyses enabled insights into how different subpopulations of students may be similar in LRI but differ in their accompanying challenge-threat orientations—and how students may differ in LRI but be similar in challenge-threat orientations. We therefore recommend that more studies assess instructional cognitive load using person-centered approaches in order to elucidate important (and sometimes quite nuanced) subpopulations of students that would otherwise be masked in variable-centered research. This will require administering instrumentation that can assess accompanying aspects of the learner. We did so via measures of inferred challenge orientation (self-efficacy, growth goals) and threat orientation (anxiety, failure avoidance goals). However, there are other indicators of challenge and threat orientations, such as affective dimensions reflecting perceived challenge-threat (e.g., enjoyment, hope, frustration, depression, anger, boredom, etc.; Pekrun, 2006).

In terms of educational practice, because the LRIS is founded on (and assesses) an instructional framework comprising five key principles, educators can be quite specifically guided in professional learning targeting these instructional principles. Martin et al. (2020a; see also Martin, 2016; Martin and Evans, 2018, 2019) have described numerous pedagogical strategies that follow from the five principles of LRI. For example, to reduce difficulty in the initial stages of learning as appropriate to the learner's prior knowledge (principle #1), they suggest pre-testing to gain a sense of where to pitch content, pre-training, and segmenting (or, “chunking”) (Pollock et al., 2002; Mayer and Moreno, 2010; Delahay and Lovett, 2019). For support and scaffolding (principle #2), suggestions include structured templates, worked examples, prompting, and advance and graphic organizers (e.g., Renkl and Atkinson, 2010; Sweller, 2012; Berg and Wehby, 2013; Renkl, 2014; Hughes et al., 2019). For sufficient practice (principle #3), deliberate practice and mental rehearsal have been recommended (e.g., Ginns, 2005; Purdie and Ellis, 2005; Nandagopal and Ericsson, 2012; Sweller, 2012). For feedback-feedforward (principle #4), corrective and improvement-oriented information has been proposed (e.g., Basso and Belardinelli, 2006; Hattie and Timperley, 2007; Shute, 2008; Hattie, 2009; Martin and Evans, 2018). For more independent and self-directed learning (principle #5), guided discovery learning has been suggested (e.g., Mayer, 2004).

There are also strategies that can foster students' challenge orientations and reduce their threat orientations. For the former, self-efficacy and growth goals were the means through which we inferred challenge orientation, and these have distinct practice implications. For self-efficacy, educators might encourage students to challenge any negative self-beliefs, especially when they are faced with difficult academic tasks (Wigfield and Tonks, 2002). Reminding students of their strengths and reiterating what they have already learned can also enhance self-efficacy (Higgins et al., 2001; Martin et al., 2019). Regarding growth goals, intervention research has demonstrated that encouraging students to set self-improvement targets (personal best goals) and teaching them how to strive to meet these targets are successful strategies (e.g., Martin and Elliot, 2016b; Ginns et al., 2018).

Anxiety and failure avoidance goals were the means through which we inferred students' threat orientation, and these also have distinct practice implications. For anxiety, there are three types of programs that tend to be offered in schools: universal programs targeting all students, selective programs targeting students at-risk of anxiety at clinical levels, and specific programs targeting students who have clinical levels of anxiety (Martin et al., 2021). Within each of these programs, cognitive–behavioral approaches tend to be successful (Neil and Christensen, 2009); here, students are specifically taught cognitive and behavioral strategies for anxiety reduction, especially for times and circumstances when anxiety is likely to strike. The use of mindfulness techniques by educators with students is another suggested strategy to reduce anxiety. In similar vein, growth mindset intervention has been found to improve individuals' stress and threat appraisals (Yeager et al., 2016). Mindfulness intervention benefits for students with negative self-beliefs have also been highlighted in several studies and reviews (Weare, 2013; Sibinga et al., 2015; McKeering and Hwang, 2019). To address students' inclination to adopt failure avoidance goals, educators are urged to reduce students' fear of failure (Covington, 2000; Martin and Marsh, 2003; Martin, 2007, 2009). Practical strategies to do this include promoting the belief that effort underpins self-improvement and does not imply a lack of ability or intelligence and making it clear that mistakes can be important ingredients for future success and do not reflect poorly on one's self-worth (Covington, 2000; Martin and Marsh, 2003).

### Limitations and Future Directions

When interpreting findings there are some limitations worth noting and which have implications for future research. First, this study relied on student reports of LRI, via the LRIS. Although the validity of this methodology has previously been demonstrated (e.g., Martin and Evans, 2018) and the psychometrics in the present study were acceptable, future research might include additional indicators such as observer ratings or self-reports by teachers to triangulate with student ratings. Second, we used the short form of the LRIS, which meant we could not estimate latent profiles on the basis of the 5 LRI principles considered separately. Future research should consider this possibility and also (using the long form) look to estimate classroom profiles (L2) characterized by different levels on these 5 principles (rather than reflecting different frequencies of the L1 profiles). For example, starting from multilevel CFA models, L2 factor scores (corrected for inter-rater disagreement) can be saved, enabling more objective ratings of the classroom. Then the L1 and L2 factor scores from this model can be used to separately estimate L1 and L2 profiles. Third, there may be instructional principles that effectively manage cognitive load on learners, but which are not in the LRI framework. To the extent additional principles are identified and can be validly assessed, we recommend including them in future research. Fourth, our data were cross-sectional which means, for example, that we were unable to determine causal ordering between the profiles and the outcomes, nor whether student and classroom profile memberships change over time. Longitudinal data and modeling (e.g., latent transition analysis) will be an important avenue in future research (Collie et al., 2020). Fifth, our study included self-efficacy and growth goals to infer challenge orientation and anxiety and failure avoidance goals to infer threat orientation. There is a need for research that assesses other indicators of challenge and threat to test the generality of our findings. For example, testing affective dimensions of perceived challenge-threat such as enjoyment, frustration, anger, boredom, etc. (Pekrun, 2006) and other challenge/approach-oriented goals such as mastery goals (Elliot, 2006) may be illuminating. There may also be potential gains in harnessing bio-psychological measures of challenge and threat in order to access real-time and more objective measures for further triangulation (Uphill et al., 2019; Martin et al., 2021). Neuro-psychological measures may additionally provide real-time indicators of experienced cognitive load. These may have the potential to deepen evaluation and understanding of LRI and its associations with challenge and threat demonstrated in this research (Berka et al., 2007; Anderson et al., 2011; Delahunty et al., 2018). Sixth, our research took place in science which is a challenging school subject (Coe et al., 2008) and one in which many students can struggle (Office of the Chief Scientist, 2014). Threat orientation may be disproportionately salient in such subjects. There is a need to explore the generalizability of our findings to other school subjects. Indeed, there is a need for research in non-science school subjects because it may be that science is more (or less) amenable to LRI. Finally, when testing for profiles in which accompanying indicators are hypothesized to be present, future research might give greater attention to real-time research methodologies. The *in-situ* dimensions of students' science engagement have been emphasized by researchers (e.g., Schneider et al., 2016) and the empirical yields of real-time engagement research has been demonstrated in other STEM subjects such as mathematics (Martin et al., 2020b).

## Conclusion

Instructional cognitive load is perceived and experienced in different ways by different students. Some students perceive cognitive load in an approach- and challenge-oriented way, while other students perceive cognitive load in an avoidant- and threat-oriented way. To better understand instructional cognitive load, it is important to assess students' experiences of this load in the context of their accompanying psychological challenge and threat orientations. The present study did so using multilevel latent profile analysis and identified numerous instructional-psychological profiles among students and also salient instructional-psychological profiles among classrooms. These profiles were further illuminated through their associations with student- and classroom-level persistence, disengagement, and achievement. The findings of this investigation have demonstrated that assessing instructional cognitive load in the context of students' accompanying psychological orientations can reveal unique insights about students' learning experiences and about important differences between classrooms in terms of the instructional cognitive load that is present.

## Data Availability Statement

The datasets presented in this article are not readily available because they are part of an industry research partnership; consent from participants to share the dataset is not available; summative data (e.g., correlation matrix with standard deviations) are available here and elsewhere to enable analyses. Requests to access the datasets should be directed to Andrew J. Martin, andrew.martin@unsw.edu.au.

## Ethics Statement

The studies involving human participants were reviewed and approved by UNSW Human Ethics Committee. Written informed consent to participate in this study was provided by the participants' legal guardian/next of kin.

## Author Contributions

AM led research design, data analysis, and report writing. PG and RC assisted with data analysis and report writing. EB and RK assisted with research design, interpretation of findings, and report writing. VM-S assisted with report writing. JP assisted with research design and report writing. All authors contributed to the article and approved the submitted version.

## Conflict of Interest

It is appropriate to note that one of the measures (the MES) in the study is a published instrument attracting a small fee (approx. US$110 per 1,000 respondents) part of which is put toward its ongoing development and administration and part of which is also donated to UNICEF. However, for this study, there was no fee involved for its use. The authors declare that the research was conducted in the absence of any commercial or financial relationships that could be construed as a potential conflict of interest.

## Footnotes

^1^There were 2,199 students in the original sample, but we removed students who did not identify their classroom (*n* = 90) as this information is necessary for multi-level analyses. Also removed were classes with fewer than 3 students, as we considered these class sizes too small to yield reliable estimates at the classroom level (viz. 38 students from 25 classes; see McNeish, 2014).
