# Supplementary material for: Assessing Instructional Cognitive Load in the Context of Students' Psychological Challenge and Threat Orientations: A Multi-Level Latent Profile Analysis of Students and Classrooms
Source: Front Psychol. 2021 Jul 1;12:656994. doi: 10.3389/fpsyg.2021.656994 (PMC8281884; doi:10.3389/fpsyg.2021.656994)

Assessing Instructional Cognitive Load in the Context of Students' Psychological Challenge and Threat Orientations: A Multi-level Latent Profile Analysis of Students and Classrooms

SUPPLEMENTARY MATERIALS

Associations Between Background Attributes and Profiles

After identifying the final student-level (L1) profile solution, the role of background attributes as predictors was examined with multinomial logistic regression, using one latent profile as a reference group. The background attributes were: age (a continuous measure), language background (0 = English speaking, 1 = non-English speaking), gender (0 = male, 1 = female), and SES based on home postcode summarized in the sample description (a continuous score, ranging from relatively greater socio-economic disadvantage to relatively greater socio-economic advantage). All results are shown in Tables S2a to S2e which provide unstandardized beta coefficients, standard errors, and odds ratios (ORs). ORs with a value greater than one indicate the increased likelihood of membership in a profile (compared with a reference profile) for every unit of increase in the predictor variable. The reverse is true for ORs less than one.

Table S1: Correlation matrix derived from the CFA that generated the factor scores for the LPAs

	Self-efficacy	Growth Goal	Anxiety	Failure Avoid Goal	Persistence	Disengage	Achieve
LRI	. 545**	.609**	-.101**	-.222*	.596**	-.646**	.180**
Self-efficacy	-	.574**	-.153**	-.266**	.708**	-.693**	.353**
Growth Goal		-	-.027	-.193**	.804**	-.692**	.213**
Anxiety			-	.548**	-.090**	.138**	-.100**
Failure Avoid Goal				-	-.272**	.347**	-.156**
Persist					-	-.761**	.316**
Disengagement						-	-.332**
Achievement							-

* $p < .05$, ** $p < .01$, LRI = load reduction instruction

Table S2a. The role of student covariates in predicting profile membership in the single-level LPA (Instructionally-Overburdened & Psychologically-Resigned as reference category)

	Instructionally-Burdened & Psychologically-Fearful			Instructionally-Supported & Psychologically-Composed			Instructionally-Optimized & Psychologically-Self-Assured			Instructionally-Supported & Psychologically-Pressured		
	b	*SE*	*OR*	*b*	*SE*	*OR*	*b*	*SE*	*OR*	*b*	*SE*	*OR*
Gender (M)	-0.068	0.225	0.934	-0.750**	0.215	0.472	-0.936**	0.269	0.392	-0.144	0.216	0.866
Age	-0.249**	0.083	0.780	-0.395**	0.080	0.674	-0.669**	0.102	0.512	-0.482**	0.083	0.618
NESB	0.109	0.483	1.115	0.056	0.453	1.058	0.213	0.501	1.237	-0.070	0.461	0.932
SES	-0.182	0.115	0.834	-0.159	0.107	0.853	0.009	0.134	1.009	-0.216*	0.108	0.806

* $p < 0.05$; ** $p < 0.01$; *b* = multinomial logistic regression coefficient; SE = standard error of the coefficient; OR = odds ratio; SES = socio-economic status; M = male; NESB = non-English speaking background

Table S2b. The role of student covariates in predicting profile membership in the single-level LPA (Instructionally-Burdened & Psychologically-Fearful as reference category)

	Instructionally-Overburdened & Psychologically-Resigned			Instructionally-Supported & Psychologically-Composed			Instructionally-Optimized & Psychologically-Self-Assured			Instructionally-Supported & Psychologically-Pressured		
	b	*SE*	*OR*	*b*	*SE*	*OR*	*b*	*SE*	*OR*	*b*	*SE*	*OR*
Gender (M)	0.068	0.225	1.070	-0.681**	0.157	0.506	-0.867**	0.218	0.420	-0.076	0.158	0.927
Age	0.249**	0.083	1.283	-0.147*	0.058	0.863	-0.421**	0.083	0.656	-0.233**	0.062	0.792
NESB	-0.109	0.483	0.897	-0.053	0.289	0.948	0.104	0.350	1.110	-0.179	0.305	0.836
SES	0.182	0.115	1.200	0.023	0.074	1.023	0.191	0.105	1.210	-0.034	0.076	0.967

* $p < 0.05$; ** $p < 0.01$; *b* = multinomial logistic regression coefficient; SE = standard error of the coefficient; OR = odds ratio; SES = socio-economic status; M = male; NESB = non-English speaking background

Table S2c. The role of student covariates in predicting profile membership in the single-level LPA (Instructionally-Supported & Psychologically-Composed as reference category)

	Instructionally-Overburdened & Psychologically-Resigned			Instructionally-Burdened & Psychologically-Fearful			Instructionally-Optimized & Psychologically-Self-Assured			Instructionally-Supported & Psychologically-Pressured		
	b	*SE*	*OR*	*b*	*SE*	*OR*	*b*	*SE*	*OR*	*b*	*SE*	*OR*
Gender (M)	0.750**	0.215	2.117	0.681**	0.157	1.976	-0.186	0.230	0.830	0.606**	0.175	1.833
Age	0.395**	0.080	1.484	0.147*	0.058	1.158	-0.274**	0.087	0.760	-0.086	0.069	0.918
NESB	-0.056	0.453	0.946	0.053	0.289	1.054	0.157	0.353	1.170	-0.125	0.328	0.882
SES	0.159	0.107	1.172	-0.023	0.074	0.977	0.169	0.105	1.184	-0.056	0.076	0.946

* $p < 0.05$; ** $p < 0.01$; *b* = multinomial logistic regression coefficient; SE = standard error of the coefficient; OR = odds ratio; SES = socio-economic status; M = male; NESB = non-English speaking background

Table S2d. The role of student covariates in predicting profile membership in the single-level LPA (Instructionally-Optimized & Psychologically-Self-Assured as reference category)

	Instructionally-Overburdened & Psychologically-Resigned			Instructionally-Burdened & Psychologically-Fearful			Instructionally-Supported & Psychologically-Composed			Instructionally-Supported & Psychologically-Pressured		
	b	*SE*	*OR*	*b*	*SE*	*OR*	*b*	*SE*	*OR*	*b*	*SE*	*OR*
Gender (M)	0.936**	0.269	2.550	0.867**	0.218	2.380	0.186	0.230	1.204	0.792**	0.226	2.208
Age	0.669**	0.102	1.952	0.421**	0.083	1.523	0.274**	0.087	1.315	0.188*	0.088	1.207
NESB	-0.213	0.501	0.808	-0.104	0.350	0.901	-0.157	0.353	0.855	-0.283	0.374	0.754
SES	-0.009	0.134	0.991	-0.191	0.105	0.826	-0.169	0.105	0.845	-0.225*	0.106	0.799

* $p < 0.05$; ** $p < 0.01$; *b* = multinomial logistic regression coefficient; SE = standard error of the coefficient; OR = odds ratio; SES = socio-economic status; M = male; NESB = non-English speaking background

Table S2e. The role of student covariates in predicting profile membership in the single-level LPA (Instructionally-Supported & Psychologically-Pressured as reference category)

	Instructionally-Overburdened & Psychologically-Resigned			Instructionally-Burdened & Psychologically-Fearful			Instructionally-Supported & Psychologically-Composed			Instructionally-Optimized & Psychologically-Self-Assured		
	b	*SE*	*OR*	*b*	*SE*	*OR*	*b*	*SE*	*OR*	*b*	*SE*	*OR*
Gender (M)	0.144	0.216	1.155	0.076	0.158	1.079	-0.606**	0.175	0.546	-0.792**	0.226	0.453
Age	0.482**	0.083	1.619	0.233**	0.062	1.262	0.086	0.069	1.090	-0.188*	0.088	0.829
NESB	0.070	0.461	1.072	0.179	0.305	1.196	0.125	0.328	1.134	0.283	0.374	1.327
SES	0.216*	0.108	1.241	0.034	0.076	1.034	0.056	0.076	1.058	0.225*	0.106	0.453

* $p < 0.05$; ** $p < 0.01$; *b* = multinomial logistic regression coefficient; SE = standard error of the coefficient; OR = odds ratio; SES = socio-economic status; M = male; NESB = non-English speaking background

Figure S1. Elbow plot for single-level LPA solutions

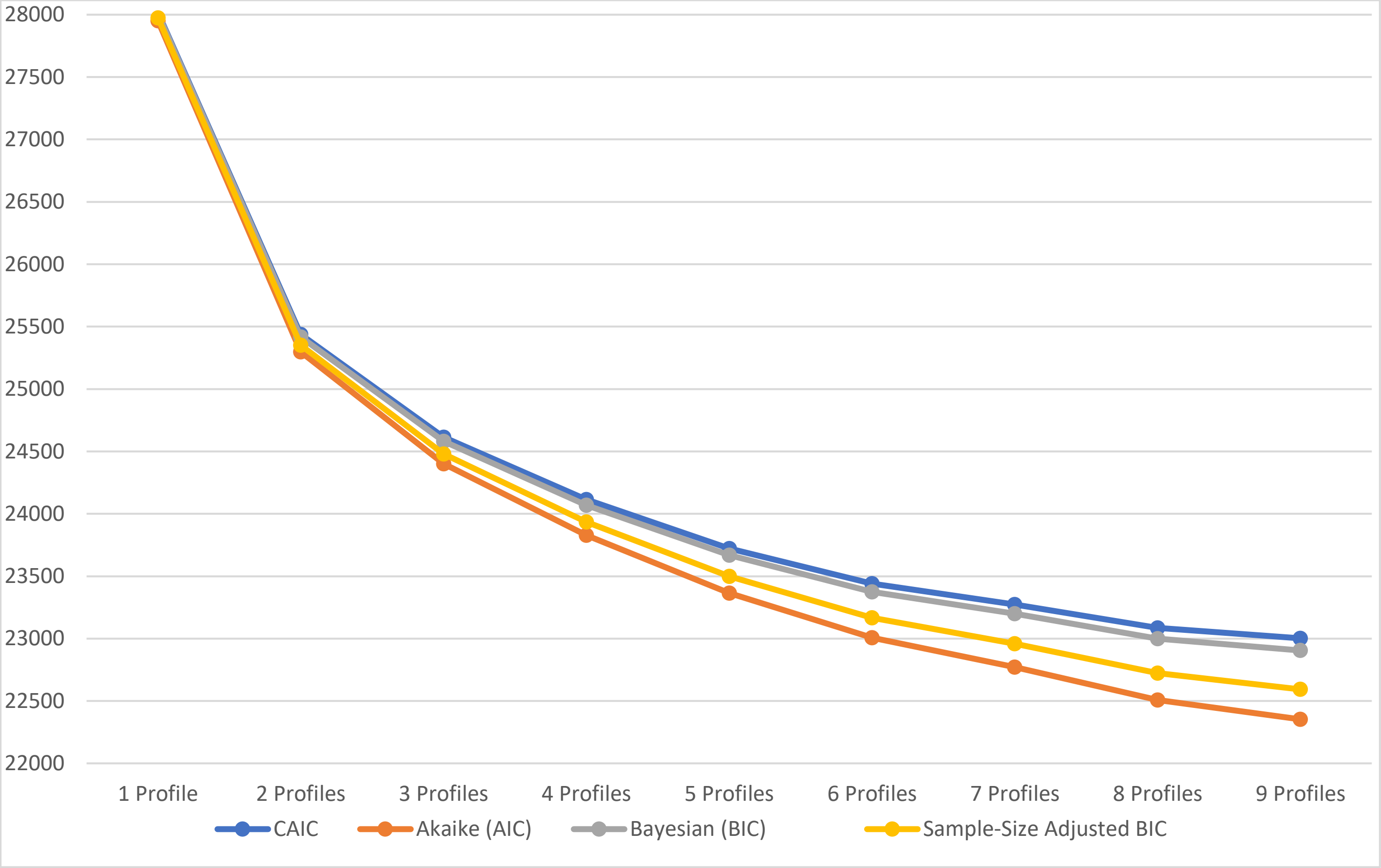

Figure S2. Elbow plot for multi-level LPA solutions

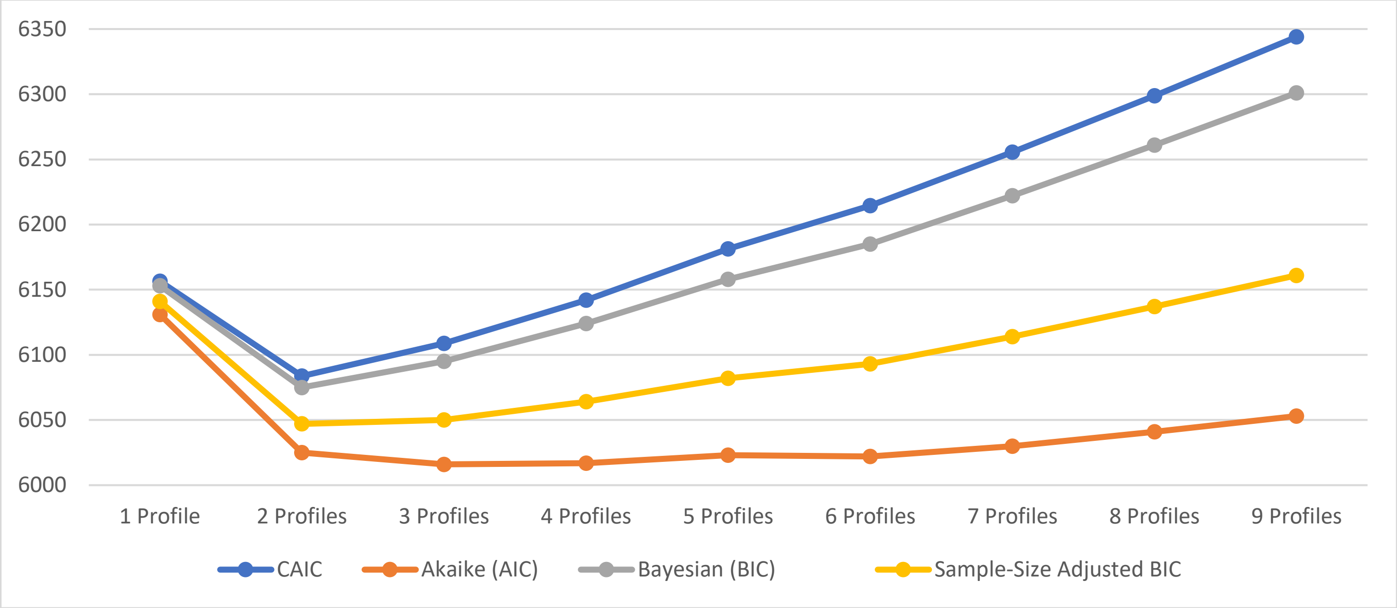

Supplement: Supplementary file 1 [file Table_1.pdf]
